# Midwives’ challenges and factors that motivate them to remain in their workplace in the Democratic Republic of Congo—an interview study

**DOI:** 10.1186/s12960-020-00510-x

**Published:** 2020-09-17

**Authors:** Malin Bogren, Malin Grahn, Berthollet Bwira Kaboru, Marie Berg

**Affiliations:** 1 Institute of Health and Care Sciences, Sahlgrenska Academy, University of Gothenburg, Arvid Wallgrens backe 1, 413 46 Gothenburg, Sweden; 2 Department of Obstetrics, Sahlgrenska University Hospital, Region Västra Götaland, Diagnosvägen 15, 416 50 Gothenburg, Sweden; 3 Swedish Embassy in Kinshasa, Democratic Republic of Congo, 93 Avenue Roi Baudouin, Kinshasa-Gombe, Democratic Republic of Congo

**Keywords:** Midwifery profession, Health workforce, Quality care, Democratic Republic of Congo, Low- and middle-income countries

## Abstract

**Background:**

The Democratic Republic of Congo (DRC) has high maternal mortality and a low number of midwives, which undermines the achievement of goal 3 of the Sustainable Development Goals (SDGs) for 2030, specifically the health of the mother and newborn. Scaling up the midwifery workforce in relation to number, quality of healthcare, and retention in service is therefore critical. The aim of this study was to investigate midwives’ challenges and factors that motivate them to remain in their workplace in the DRC.

**Methods:**

Data were collected in two out of 26 provinces in the DRC through ten focus group discussions with a total of 63 midwives working at ten different healthcare facilities. Transcribed discussions were inductively analysed using content analysis.

**Results:**

The midwives’ challenges and the factors motivating them to remain in their workplace in the DRC are summarised in one main category—*Loving one’s work makes it worthwhile to remain in one’s workplace, despite a difficult work environment and low professional status*—consisting of three generic categories: *Midwifery is not just a profession; it’s a calling* is described in the subcategories Saving lives through midwifery skills, Building relationships with the women and the community, and Professional pride; *Unsupportive organisational system* is expressed in the subcategories Insufficient work-related security and No equitable remuneration system, within Hierarchical management structures; and *Inadequate pre-conditions in the work environment* includes the subcategories Lack of resources and equipment and Insufficient competence for difficult working conditions.

**Conclusion:**

Midwives in the DRC are driven by a strong professional conscience to provide the best possible care for women during childbirth, despite a difficult work environment and low professional status. To attract and retain midwives and ensure that they are working to their full scope of practice, we suggest coordinated actions at the regional and national levels in the DRC and in other low-income countries with similar challenges, including (i) conducting midwifery education programmes following international standards, (ii) prioritising and enforcing policies to include adequate remuneration for midwives, (iii) involving midwives’ associations in policy and planning about the midwifery workforce, and (iv) ensuring that midwives’ working environments are safe and well equipped.

## Background

Healthcare provided by midwives who are educated and regulated according to global professional standards is defined as a core strategy for decreasing maternal mortality rates and improving reproductive, maternal, and newborn health [1–3]. However, a shortage of professional midwives informed by international standards is detrimental to the need for universal access to high-quality care [1]. This is especially a challenge in Sub-Saharan Africa, for instance, in the Democratic Republic of Congo (DRC), which reports a maternal mortality rate of 846 per 100,000 live births [4] and a fertility rate of 6.2 [5]. These numbers can be explained by, among other things, poor healthcare quality [6], which in turn is partially due to a severely strained health system afflicted by many years of political conflict [7]. With only one midwife per 20,000 persons, the midwifery workforce in the DRC is far from the minimum required for meeting the predicted need for midwives within maternity care [8], and for reaching Sustainable Development Goal (SDG) 3 on health aiming at a maternal mortality rate of fewer than 70 per 100,000 live births by 2030 [9].

However, scaling up midwifery healthcare implies not only increasing the quantity of midwives, but also customising evidence-based healthcare to fit local conditions and necessities [10, 11]. Thus, investigating what enables midwives to provide quality care is crucial [5].

In the DRC, the midwifery profession has received increased attention only in recent years [9]. Since the country’s educational reform in 2013, two paths of midwifery education are offered at the higher education institutes for applied medical sciences (ISTM). Both lead to an advanced diploma. The first, a 3-year direct-entry full-time programme, is currently offered at 16 ISTMs located in 12 of the country’s 26 provinces. The second is a 12-month programme for nurses educated within a 3-year programme at a higher academic level. Both programmes consist of 60% theory and 40% clinical practice. This programme, while too short to fulfil international standards, is currently offered at three ISTMs located in three provinces [12]. As of 2019, a total of 3646 midwives had been schooled in the new midwifery educational programmes. However, despite the educational reform in 2013, the midwifery educational system faces several challenges. These include a low academic level among the tutors within the programmes, including the midwifery educators and clinical preceptors. Furthermore, the midwifery profession is not yet regulated and is thus not allowed to work as an autonomous profession within a defined area of practice and competence [9, 13]. This means that after completing an education programme, midwives are often obliged to work in areas that are not related to the midwifery area of practice [9].

This situation for midwives in the DRC echoes findings from a global consultation with 2470 midwives in 93 countries (not including the DRC). Hierarchies of power and gender discrimination were identified as barriers to providing high-quality, respectful care for women, newborns, and their families [14]. In low- and middle-income countries, such barriers have been identified to include economic, social, and professional areas [15–17]. Contributing factors include gender inequalities with a lack of power for women [15, 17], low professional autonomy [14, 16], and unmanageable workloads [16], the last of which is also identified as a significant barrier in high-income countries [18].

In a conceptual model on how health worker motivation is influenced and how health sector reform can positively affect worker motivation, it has been concluded that motivating factors have a high impact on health worker performance and on general health outcomes in health workers and that the factors cover both individual, organisational, and cultural dimensions [19]. Creating a motivating work environment in alignment with midwives’ preferences and well-being is identified as crucial for avoiding the unnecessary resignation of midwives, and thereby increasing the possibility to achieve universal access to sexual, reproductive, maternal, and newborn care [11, 20, 21].

To secure high-quality care before, during, and after childbirth, it is essential to understand not only what challenges midwives face but also what motivates them to remain in their profession [14, 15]. In accordance with the recommendations by the United Nations Population Fund in East and Southern Africa [13], there is a need to understand what encourages midwives in their work and enables them to provide quality care. There is abundant research from high-income settings on what challenges midwives face and factors that force them to leave the profession, but similar research from low- and middle-income settings is much more limited. The aim of this study was to investigate midwives’ challenges and the factors motivating them to remain in their workplace in the DRC, in order to identify strategies that enable the DRC to use professional midwives in an optimal way in its strive to reach the 2030 SDGs associated with maternal and newborn health.

## Methods

### Study design and setting

A qualitative study including focus group discussions (FGDs) with midwives was conducted. This method is appropriate to use when seeking a contextualised understanding within a topic on which little is known [22].

The healthcare system in the DRC is divided into health zones and offers care at three levels: the primary level at healthcare centres, some of which include maternity wards; the secondary level at district hospitals with the capacity to perform caesarean sections; and the tertiary level at referral hospitals. Our study was conducted at selected healthcare facilities, including both public and private settings, in the two provinces of Maniema in eastern DRC and Kasai in the central part of the country, which have a high incidence of maternal and newborn death and are part of a larger health-strengthening project—BOMOYI—with a focus on maternal and newborn health, managed by the local organisation Soins de Santé Primaires en Milieu Rural (SANRU Asbl).

One of the weaknesses of the public health system in the DRC is a poorly structured remuneration system for public servants. The pay structures for midwives in the DRC vary. The public sector pay is commonly composed of different elements, with a basic salary as a small component of the overall pay. In order to be on the public service payroll, a civil service number is required, which many midwives do not have. However, according to anecdotal information, a majority of midwives who do have a civil service number nonetheless do not receive regular payment. The monthly salary (as of 2020) ranges from 80,000 to 150,000 Congolese Francs (US$ 40–75). Allowances, taken from resources levied through patient fees, contribute more to a midwife’s total income than salary does. More than 40% of healthcare expenditures in the DRC are basically supported by households [23]. The cost of normal childbirth varies depending on the level of care, place of residence, whether the care is provided at a private or public facility, and whether the healthcare facility receives external funding. In general, at the primary level, the cost of normal birth is US$ 10–20 and at the secondary level US$ 15–25, while at referral hospitals, the estimated cost is US$ 20–35.

### Participants and data collection

Managers at the selected healthcare facilities representing all three levels were informed about the study. All of them approved the study and provided contact information for midwives working at different maternity units, who were then invited to participate by SANRU focal staff. Interested midwives were given verbal and written information about the study, including the fact that participation was voluntary and that withdrawal was allowed at any time without explanation. Midwives agreeing to participate signed an informed consent.

Ten FGDs were conducted in October 2019, at ten healthcare facilities near the midwives’ workplaces. Six to eight midwives participated in each FGD, totalling 63 midwives who worked at different healthcare facilities representing both governmental and private sectors, all of which were integrated into the DRC’s public healthcare system. For details about the study participants, see Table 1. Two of the authors (MBo and MBe) conducted the FGDs, together with a representative from the national midwifery association who acted as an observer. The FGDs were audio-recorded and lasted 30–45 min, with a mean of 40 min. MBe led the discussion in French based on an interview guide (see Additional file 1) and translated continuously during the FGD into Swedish to MBo, who made field notes and asked clarifying questions. The audio-recorded FGDs were transcribed from French into Swedish text.

**Table 1:** Characteristics of study participants (*n* = 63)

Variable	Midwives, ***n*** (%)
***Gender and age***	
Male	14 (22%)
Female	49 (78%)
Age range, years	28–67
Mean age, years	42
***Professional education***	
Midwife 3 years direct entry	53 (84%)
Nurse-midwife (1 year post-nursing programme)	1 (2%)
Auxiliary nurse-midwife	9 (14%)
***Highest level of completed academic qualification***	
Bachelor’s degree in either obstetrics, education and administration, public health, or community health/epidemiology	13 (21%)
No academic degree	50 (79%)
***Years working as a midwife***	
Range	0–46
Mean	9

### Data analysis

The data were analysed through an inductive approach according to content [24]. First, the text was read through several times to become familiar with the data. Next, in new readings, meaning units were marked, compared, and sorted into codes, which were then compared and clustered into subcategories, generic categories, and one main category. After several analysis refinements moving from text details to wholeness, the results were finalised. An example of the analysis process is shown in **Table** **2**.

**Table 2:** Example of an analysis process from a unit of analysis to generic category

Unit of analysis	Open code	Subcategory	Generic category
It is our gift to do this, to help the population, to help the women.	Helping the people	Building relationships with the women and the community	Midwifery is not just a profession; it’s a calling
Love for our country and the population is a strong motivating factor for our work.	Love for the people		
We want to receive a good salary, a fair salary for the work we do.	Lack of appropriate payment for the work	No equitable remuneration system	Unsupportive organisational system
We receive US$ 5/month. We cannot live on our salary; we have to work the fields on our days off.	Salary insufficient for daily life		

## Results

The midwives’ challenges, and the factors motivating them to remain in their workplace in the DRC, are summarised in one main category—Loving one’s work makes it worthwhile to remain in one’s workplace, despite a difficult work environment and low professional status—consisting of three generic categories: *Midwifery is not just a profession; it’s a calling*, comprising the three subcategories (1) Saving lives through midwifery skills, (2) Building relationships with the women and the community, and (3) Professional pride; *Unsupportive organisational system*, comprising the three subcategories (1) Insufficient work-related security, (2) No equitable remuneration system, and (3) Hierarchical management structures; and *Inadequate pre-conditions in the work environment*, comprising the two subcategories (1) Lack of resources and equipment and (2) Insufficient competence for difficult working conditions (Table 3).

**Table 3:** Summary of results

Main category: Loving one’s work makes it worthwhile to remain in one’s workplace, despite a difficult work environment and low professional status.	
Generic categories	Subcategories
Midwifery is not just a profession; it’s a calling	Saving lives through midwifery skills
	Building relationships with the women and the community
	Professional pride
Unsupportive organisational system	Insufficient work-related security
	No equitable remuneration system
	Hierarchical management structures
Inadequate pre-conditions of the work environment	Lack of resources and equipment
	Insufficient competence for difficult working conditions

### Midwifery is not just a profession; it’s a calling

The generic category *Midwifery is not just a profession; it’s a calling* concerns the midwives’ motivation in their work, which included saving lives through midwifery skills, building relationships with the women and the community, and having professional pride.

#### Saving lives through midwifery skills

Saving the lives of mothers and newborns was a strong motivating factor. Specifically, it was motivational to have skills within the midwifery domain, such as managing the full continuum of care during pregnancy and labour, supporting women in having normal physiologic births, or being able to handle complications:

I feel very good when I can handle the complications during a birth and afterwards; for example, if a woman comes in unconscious and I can handle it. (FGD1)

The midwives felt that their main role was to save lives and thereby contribute to decreasing the country’s high maternal mortality rate. Saving a woman’s life was valued as a measurement of high-quality care. This was a driving force in their daily work and made them feel motivated, as their work made a difference for the survival of women in the DRC:

I have given my life to save lives (FGD3) // We have decided to always be at the women’s side, to save their lives. We cannot break this promise to save their lives. (FGD2) //I want to continue working at my workplace to be able to make a difference and decrease the maternal mortality. (FGD4)

#### Building relationships with the women and the community

Building relationships with the women and playing a significant role in the community were central to and valued by the midwives. They repeatedly expressed having strong feelings of love for the women and for the community and experienced their work as important and meaningful:

It’s the love for the sick that motivates us. (FGD7) // I want to continue this work because of the love for our country and the population. (FGD1)

Mutual trust and a duty to the community characterised the midwives’ perceptions of their relationships with the women. They felt appreciated and trusted by both the women and the community. They emphasised that they wanted to continue their work in order to be loyal to their patients and to society as a whole:

I feel good when the woman has trust in us … she surrenders herself in confidence to us. (FGD6) // We have built trust with the women and made them feel safe. (FGD5) // If we abandon our work, we leave a people that will suffer. (FGD2)

#### Professional pride

The midwives radiated professional pride; they had a profession that they enjoyed and believed in. Their position in society to help women also gave them feelings of satisfaction and accomplishment:

We have studied to work as midwives. We are proud of our profession, to help women. (FGD4) // The greatest motivation is that we are midwives and we love the profession. (FGD1)

The midwives felt that their role as midwives was essential because of their ability to help women. This resulted in feelings of commitment, duty, and resilience, despite their difficult working conditions, which in turn characterised their sense of professional identity and gave them motivation. Sacrificing themselves for the belief in their profession was motivational, in the sense of making a difference for the women:

Because we are midwives, we have to continue helping the women. We are midwives whether or not we are motivated. (FGD2) // It is our profession! We have chosen our profession and must continue. We have committed ourselves and will continue to be midwives, regardless of the situation. This is our calling; we exist to work as midwives. (FGD6)

### Unsupportive organisational system

The generic category *Unsupportive organisational system* describes how midwives were challenged by insufficient work-related security, hierarchical management structures, and the lack of an equitable remuneration system.

#### Insufficient work-related security

The midwives’ work environment was surrounded by insecurity. Especially during night shifts, due to the darkness, they often felt frightened on their way to and from work. Many years of conflict and continuous unstable security in society had installed fear in them, and they were afraid of being a victim of aggression, including rape. Practical constraints increased their insecurity, such as the lack of transportation to and from work and the inability to pay for secure transport such as a taxi or a motorbike, or not having a flashlight or money to pay for batteries, further amplified their feelings of insecurity and fear:

Working at night makes us insecure… we are always afraid in the dark. (FGD3) // We are all scared of the military, scared of being raped… The conflict was in 2018 and there were two doctors and midwives who were killed then. (FGD2)

A professional identity card could protect them, but not all midwives had received such a card from their employer:

We bring ID card to work. When the military comes and we can show our ID card, there are no approaches from the military. (FGD1)

#### No equitable remuneration system

There was no equitable remuneration system in place, and the midwives stressed the lack of regular payment or compensation for their employment. There was no fixed amount of money for their services that was paid on a monthly basis. Some expressed that their salary was too low and was not sustainable for them to live on, while others described having more of a monetary incentive, equal to US$ 5, than any actual salary. At some facilities, they were dependent on the patient fees in order to get paid. This meant that sometimes the midwives had to take up a second job in order to meet their everyday needs, which resulted in feelings of increased everyday stress and being underappreciated:

We cannot live on our salary. We work shifts and then have to work in the fields on our days off. (FGD6) // We don’t receive any income from the state, only through patient fees. Our work is voluntary work, we don’t even get soap. (FGD9)

#### Hierarchical management structures

Another challenge involved hierarchical management structures within the healthcare facility. Several midwives expressed feeling insecure and uncertain in their employment and their relationships with their supervisors. Many midwives had no employment contract, while others could be punished for, for example, being late to work by losing their job or being sent home for 3 months without work or payment. Not feeling safe in their job caused the midwives to feel uncertain in their everyday work situation:

We cannot complain, we lose our job if we complain… We have never signed anything when we started our employment here, we don’t have any contracts. (FGD4) // If you question the supervisor you can be let go without pay for three months… So I don’t say anything, because then I won’t get paid. (FGD1) // I am afraid that I won’t get to work on time, which can be due to not being able to find a motorbike or not being able to pay for it. (FGD4)

A few midwives who worked at a private healthcare facility did not have such an unsecured working condition. They could even question work routines without being afraid of losing their job.

Many midwives reported being placed at departments where their specific midwifery professional competence was not useful, such as surgery/internal medicine wards, the pharmacy, or the administration office. They could be moved around according to their supervisor’s preferences rather than their specific domain. Not being allowed to practise their profession and not receiving recognition for their work made it very demanding to continue their job, leading the midwives to feel frustrated and undervalued. A need for support from the local midwifery association was expressed:

They don’t make use of our potential as midwives; we have to work in other departments like medicine, paediatrics, surgery… I feel like I’m used as an object. (FGD4) // We want the state to care about midwives. (FGD6)

### Inadequate pre-conditions in the work environment

The generic category *Inadequate pre-conditions in the work environment* describes how the midwives were challenged by inadequate pre-conditions at work, including a lack of resources and equipment as well as insufficient competence for difficult working conditions. The midwives simply lacked the means to carry out their work and provide quality care.

#### Lack of resources and equipment

For the midwives, a lack of resources and equipment to perform their daily work duties posed a significant challenge. This problem concerned several areas, such as space and function and a shortage of the basic, essential clinical equipment needed to provide care during labour, birth, and after. This could include a lack of birth sets, syringes, caesarean kits, blood pressure cuffs, oxygen, medicines, and vacuum extractors. There was also a shortage of personal protective equipment like work uniforms, visors, gloves, boots, soap, birth caps, and disinfectant. This caused the midwives to feel worried about being infected with communicable diseases. Working without sufficient resources impaired not only the safety of their work environment but also their sheer ability to carry out work of sufficient quality:

We have not received any external support to rebuild the hospital after the war… We’re working under tough conditions. We only have one birthing set… one vacuum extractor, but it does not work. (FGD7) // I feel bad when we have learned different techniques like vacuum extraction and revival, but cannot perform these due to a lack of material… We want work clothes; we have to pay for them ourselves. We need boots to protect us, and medicines. (FGD3)

The facilities were described as inadequate for meeting the needs of the number of patients coming to the clinics. A lack of birthing beds could mean that two to three women had to share one bed. There was often a shortage of electricity, which led to a lack of lighting and the incubator and revival machines not functioning. Lacking materials and having inadequate facilities to work in served as a constant source of frustration for the midwives, and was a major challenge to their ability to provide care.

#### Insufficient competence for difficult working conditions

The midwives expressed having insufficient competence in providing care in critical conditions, such as resuscitation of mother and child, the third stage of labour, and the postpartum period. They described thanking God if things went well, or hoping for good outcomes, rather than trusting their competence to handle the possible complications. Another tough working condition was when poor, dirty women, for instance, sex slaves, came to the hospital seeking care. This often caused the midwives to feel frustrated, wishing they had more resources to help these women. The working conditions led to the midwives often feeling tired after work and not having the time or means to eat. Insufficient competence, in combination with difficult working conditions, resulted in their undertaking an overwhelming personal responsibility and feeling inadequate at work. The midwives often brought these feelings home, which led to negative effects on their general well-being:

I feel bad when mother and child die… or when a birth ends with excessive bleeding… If I can’t see that I have done enough I blame myself. This effects how I feel at home, can disrupt my sleep. (FGD5)

To manage the difficult situations described above, the midwives stressed needing additional competence through proper, sufficient education and continual professional development. They felt that they needed more education and training in all areas in order to increase their general competence, as their skills were insufficient. This would result in increased confidence as midwives, and better outcomes in their work. Due to the education reform of 2013, they felt an increased need to update their knowledge. However, training was not incorporated in all working environments and, when it was provided, it often did not include everyone, which led to unequal conditions and stagnation of the midwives’ competence:

We have the old education system and because of the new education reform we need to be updated in the new educational programme. We lack competence in how to support during labour, how to avoid bleeding… (FGD8) // We really need further education in order to increase our professional capacity. We do not receive continual training. (FGD2)

## Discussion

In this qualitative interview study with midwives working in two of the 26 provinces of the DRC, the results show that the main force motivating and driving the midwives to work is a strong feeling of love for their work. Their working conditions are extremely difficult. However, they regard midwifery as not just a profession but a calling, which makes the job worthwhile. In tune with what was recently found in the global consultation mentioned earlier, they are generally very committed to providing care to women and newborns due to a high professional conscience and loyalty to their patients, despite their tough working environments [14].

The DRC midwives in our study were motivated to continue working and building relationships with the women and the community, driven by feeling a strong professional pride and having the skills to save lives. On the other hand, they faced challenges in the remaining of their work due to factors such as an unsupportive organisational system, with inadequate pre-conditions in the work environment. This is in line with a conceptual framework on health worker motivation presented by Franco et al. [19], which defines key determinants influencing workers motivation at individual, organisational, and cultural and community levels.

Among the challenging work factors that the DRC midwives in our study experienced was an unsupportive organisational system, which was linked to insufficient work-related security, a lack of a proper remuneration system, and hierarchical management structures. Filby et al. have concluded that such factors are the result of the lack of status of, and respect for, the midwifery profession. This in turn puts midwives’ occupational health and safety at risk [15].

The lack of an equitable remuneration system and insufficient security when going to and from work that was experienced by the DRC midwives in our study has also been confirmed to be highly problematic in other studies in low- and middle-income settings [15, 16], leading to serious challenges in managing daily life and accentuating the profession’s low status. In settings where salaries are extremely low or unpredictable, proper remuneration is seen as crucial to worker motivation [25–27].

The World Health Organization (WHO) emphasises work security, proper remuneration, and professional health and safety as core dimensions of strengthening the health workforce and ensuring decent employment terms. This can serve as guidance for health systems in general, in order to build more reliable and supportive organisational systems [28].

With regard to remuneration in particular, the midwifery workforce in the DRC, as was also found in the global consultation of midwives, suffer from insufficient and irregular remuneration [14]. This corresponds to staff within weaker professions in the DRC who also work without being on a payroll, and thus depend on fees levied by health facilities on patients. In contrast, it has been reported that there are many cases of ghost workers, i.e. people who are paid but are non-existent in the health services [29]. According to a recent study by WHO, this could be detected and prevented through increased transparency and accountability in payroll processes, improved recordkeeping and strong record management systems, monitoring of human resources for health, and the use of specific technology tools [30]. Removing such ghost workers could result in substantial savings that could be used to improve the remunerations of working healthcare professionals.

For midwives in the DRC and elsewhere, to be able to provide their full scope of midwifery practice informed by the International Confederation of Midwives [31], and thereby contribute to improved health outcomes for women, newborn, and families [1], it is of critical importance that they are deployed within their specific professional area, i.e. sexual, reproductive, and maternal healthcare. The midwives in our study described being moved around by management to different departments far from their competence area, which resulted in their competence not being fully used. It also made them feel devalued and underappreciated. This is not unique to midwives in the DRC; however; midwives in many parts of the world experience that there is a poor understanding and use of their professional competence, and they often feel undervalued [14–16].

Strengthening the midwifery association has been identified as one way to improve the status of the midwifery profession. This was also mentioned by the midwives in our study. It is known that ensuring strong midwifery associations, and thereby establishing autonomy and recognition for the profession, is imperative for raising midwives’ status and enabling them to provide quality care [14]. Thus, in accordance with a global study conducted by Lopes et al. [32] including 73 midwives’ associations representing 67 countries, it is also necessary for the DRC to strengthen the country’s midwifery association and to include midwives in policy discussions concerning the profession—which, although it is stable and recognised by the government, still struggles to ensure education, regulation, and respect for the autonomous profession [9].

Inadequate pre-conditions in the DRC midwives’ work environment was another challenge found in our study. The midwives lacked both functioning healthcare facilities and materials, which added to their already difficult work situation. Constantly having a shortage of resources and equipment prevented them from working safely and jeopardised their provision of quality care. Lacking safety equipment is known to make midwives more vulnerable to infectious diseases [15], and having appropriate facilities and sufficient resources is regarded as necessary for worker motivation and performance [28]. Therefore, it is important that health systems improve the availability of resources and functioning facilities to fit the care services, in order to enable midwives to provide care of high quality.

The midwives in this study expressed a need for additional professional competence as part of their continuing professional development. The education system for midwives in the DRC is known to need significant improvement [9] and if reformed could increase both the capabilities and status of the profession. Already burdened by a high maternal mortality rate and heavy workloads, and with inadequate competence, time, and resources to do more than the absolute necessary work, the midwives in this study were left feeling frustrated and less motivated. These findings correspond to the global consultation [14], in which midwives experienced difficult work situations due to heavy workloads and shortage of staff, high levels of maternal and newborn mortality rates, and a lack of sufficient competence to autonomously manage work tasks, which made them feel frustrated, guilty, and inadequate. As such, this can contribute to distress and burnout, which in turn prevents midwives from being able to provide quality care and can eventually cause them to leave the profession [15]. This phenomenon does not seem to differ between settings in high-, middle-, and low-income countries [16, 18]. Therefore, in order to retain midwives in their work positions and enable them to provide quality care, it is crucial to create supportive work environments by ensuring sufficient pre-conditions [33]. Hence, midwives need to take the power to influence their own situation [15]. When midwives are included in customising their work environments, it has proven to result in improved quality of care for women and newborns around the globe [14], and it is suggested that the DRC also embraces this.

### Methodological considerations

This study has limitations. Two that particularly stand out are common criticisms of FGDs when it comes to obtaining valid data: (i) the possibility that the participants may not have expressed their honest and personal opinions about the topic under discussion and (ii) compared to individual interviews, there is no guarantee of depth in the topic being discussed [34]. However, we chose to conduct FGDs to encourage a discussion among the participants that would enable them to be inspired by each other and continue with reflections stimulated by each other’s statements. Our conviction is that this methodology favoured openness as it enhanced a sense of community, sharing common problems. We therefore ensured that everyone could express their opinions through the open questions and by being attentive to allow everyone the opportunity to talk. The number of participants per group—six to eight—enabled a good discussion climate and ensured that everyone’s views were heard. A strength was that the participants represented different healthcare facilities, both public and private, and different regions, and thereby offered deeper and more varied experiences and reflections. Despite that the interviews were conducted in only two out of 26 provinces, we find them valuable as a predominant strength of the study is that the working environment of the midwives in the DRC has not been made visible in earlier studies. In general, there is a shortage of recent studies on what motivates midwives in their work globally, which this study addresses. It is worth noting that the findings regarding the challenges and motivating factors for remaining in their workplace mentioned by the midwives in this study are context-specific and that different countries and settings must interpret these in light of their own context. The first and last authors (MBo and MBe) are Swedish researchers and midwifery experts and are both familiar with the DRC context and with conducting research in low-income settings. In this study, being a foreigner proved to be a strength in the sense that the participants were keen to explain their challenges and factors that motivate them to remain in their workplace in the DRC to someone who was not from there. The third author (BK), who grew up in the DRC and is a researcher and an expert on systems strengthening, took part in the steps of the research process and acted as an outsider in the interpretation of the results.

## Conclusion

The findings of this investigation of challenges and factors motivating DRC midwives to remain in their workplace should be of serious concern to the profession and its leaders. Midwives in the DRC are driven by a strong professional conscience, and their provision of care for women in childbirth depends on their feeling that their professional work is a calling. To overcome an unsupportive organisational system and inadequate pre-conditions in the working environment, which hinders midwives in providing high-quality care, in accordance with the 2020 Triad Statement made by the International Council of Nurses, the International Confederation of Midwives, and the World Health Organization [35], it is suggested that policymakers, employers, and regulators in the DRC and settings with similar conditions coordinate actions in the following:

✓ Increasing funding to educate midwives, informed by international standards

✓ Prioritising and enforcing policies to include adequate remuneration to attract and retain midwives within midwifery practice

✓ Involving midwives’ associations in policy and planning concerning the midwifery workforce

✓ Ensuring that midwives are working to their full scope of practice

✓ Ensuring that midwives’ working environments are safe and well equipped (functional facility with water, supplies and commodities, communication and transportation for referral)

✓ Providing an enabling environment for midwifery care to improve conducive working conditions that enable midwives to provide respectful care

## Supplementary information

**Additional file 1:.** Interview guide.

## Data Availability

Available upon request.

## Abbreviations

DRC: Democratic Republic of Congo
SDG: Sustainable Development Goal
ICM: International Confederation of Midwives
SANRU: Soins de Santé Primaires en Milieu Rural
FGD: Focus group discussion
WHO: World Health Organization
