# Supplementary material for: Midwives’ challenges and factors that motivate them to remain in their workplace in the Democratic Republic of Congo—an interview study
Source: Hum Resour Health. 2020 Sep 17;18:65. doi: 10.1186/s12960-020-00510-x (PMC7499901; doi:10.1186/s12960-020-00510-x)
Supplement: Supplementary file 1 — Additional file 1:. Interview guide. [file 12960_2020_510_MOESM1_ESM.docx]

**Appendix 1**: Interview Guide

1. Can you tell me what makes you feel good at work?
2. What makes you want to continue working at your workplace?
3. Can you tell me about any obstacles you face working as a midwife at your current workplace?
4. Do you feel safe and secure in your working environment? Please expand on this!
5. What needs to be improved in your working environment to address these problems/issues?
6. How would you say you feel when you have finished a work shift (Ex: Exhausted, Traumatized, Angry, Impatient, Happy, Fulfilled, At ease, Scared, Incompetent, Lonely or alone, I want to leave the profession, I want to find a job in another country)?
7. Are there any obstacles that prevent you from providing optimal care? Please give examples.
